# Should we use angiotensin II infusion in COVID-19-associated vasoplegic shock?

**DOI:** 10.1186/s13054-020-03144-6

**Published:** 2020-07-09

**Authors:** Karim Bendjelid

**Affiliations:** 1 Cardiovascular Unit, Intensive Care Division, University Hospitals, Geneva, Switzerland; 2 Geneva Hemodynamic Research Group, Geneva, Switzerland; 3 Faculty of Medicine, Geneva, Switzerland

**Keywords:** ACE2, SARS-CoV-2-ACE2, Angiotensin

To the editor:

We read with great interest the recent research letter, published in *Critical Care*: “Angiotensin II infusion in COVID-19-associated vasodilatory shock: a case series”, by Zangrillo and collaborators [1]. In the referenced review, the authors recommended the use of angiotensin II (ANGII) infusion in COVID-19-associated vasoplegic shock. Moreover, the authors, who studied a cohort of consecutive ventilated patients in COVID-19-dedicated ICUs, suggested the use of ANGII as a primary vasopressor in this setting [1]. In this regard, we would like to discuss the present results and opinion.

COVID-19 is expected to be a challenge in ICUs globally until a safe and effective treatment is found. We agree that efforts to study the disease are paramount to advance our understanding of the disease and to improve treatment options. However, the rationale of the use ANGII infusion in the present viral sepsis [2] is difficult to understand, as there is a reduction in pulmonary angiotensin-converting enzyme 2 (ACE2) expression related to SARS-CoV-2 infection [3]. Indeed, a consensus of evidence from various studies favours a primary role of ACE2 in efficiently degrading ANGII to Ang-1-7 [3]. Consequently, the loss of ACE2 shifts the system to an overall higher ANGII level due to the impaired ability of ACE2 to degrade ANGII.

The present fact may explain the relatively spectacular haemodynamic stability of patients with COVID-19, even in deeply sedated mechanically ventilated patients, with a tendency towards a hypertensive profile during the weaning stage. When used, the dose of norepinephrine is very modest (less than 0.1 g/kg min) in critically ill patients with COVID-19. Finally, Liu et al. recently reported that the circulating levels of ANGII were significantly higher in patients with COVID-19 than in healthy controls [4]. In this regard, should we infuse ANGII in patients with already high plasmatic levels of ANGII?

The present question is very important, as ANGII is acknowledged as a promotor of acute lung injury and ARDS induced by coronavirus [5]. The mortality of patients with COVID-19 being related to ARDS and respiratory failure, we need to be cautious when discussing therapy for patients with COVID-19 in shock, as the primary organ that is threatened is the lung.

## Authors’ response

Alberto Zangrillo, Giovanni Landoni, Luigi Beretta, Federica Morselli, Ary Serpa Neto, Rinaldo Bellomo

We thank Prof. Bendjelid for their interest in our recent letter published in *Critical Care*. The major concern raised was the rationale of the use of angiotensin II infusion in the present COVID-19. To enter in the cell, the SARS-CoV-2 uses the angiotensin-converting enzyme (ACE) 2 and the cellular protease TMPRSS2 [6]. The S protein of the virus is able to bind to ACE2 with greater affinity than the SARS-CoV-1, and this can explain the increased transmission in the current pandemic [7]. In addition, it is suggested that the infectivity is closely correlated with the degree of ACE2 expression [6]. Thus, any intervention or strategy aiming to decrease the ACE2 expression could decrease the impact of the SARS-CoV-2 infection.

The ACE2 is responsible for the degradation and hydrolysis of angiotensin II into angiotensin-(1-7) [8]. The angiotensin II could also then compete with the SARS-CoV-2 for the ACE2 receptor. In addition, angiotensin II can cause the internalization and downregulation of ACE2 through its binding to the AT1 receptor [9]. As recently reported, the competitive inhibition, downregulation, internalization, and then degradation of ACE2 may decrease the degree of viral spread [8].

Given the above considerations, the support of mean arterial pressure with angiotensin II in preference to other vasopressors in the setting of SARS-CoV-2 infection seems physiologically rational. However, we agree that well-powered designed randomized clinical trials are necessary to draw firm conclusions on the effect of angiotensin II on patient-centred outcomes.

## Data Availability

Not applicable.
